# Differentiating Pressure Ulcer Risk Levels through Interpretable Classification Models Based on Readily Measurable Indicators

**DOI:** 10.3390/healthcare12090913

**Published:** 2024-04-27

**Authors:** Eugenio Vera-Salmerón, Carmen Domínguez-Nogueira, José A. Sáez, José L. Romero-Béjar, Emilio Mota-Romero

**Affiliations:** 1 Servicio Andaluz de Salud, Distrito Sanitario Granada-Metropolitano, Centro de Salud Dr. Salvador Caballero de Granada, 18012 Granada, Spain; eugenio.vera.sspa@juntadeandalucia.es (E.V.-S.); emilio.mota.sspa@juntadeandalucia.es (E.M.-R.); 2 Instituto de Investigación Biosanitaria (ibs.GRANADA), 18014 Granada, Spain; 3 Inspección Provincial de Servicios Sanitarios, Delegación Territorial de Granada, Consejería de Salud y Familias de la Junta de Andalucía, 41071 Sevilla, Spain; mcarmen.dominguez@juntadeandalucia.es; 4 Department of Statistics and Operations Research, University of Granada, Fuente Nueva s/n, 18071 Granada, Spain; joseasaezm@ugr.es; 5 Institute of Mathematics, University of Granada (IMAG), Ventanilla 11, 18001 Granada, Spain; 6 Department of Nursing, University of Granada, Avda. Ilustración 60, 18071 Granada, Spain

**Keywords:** pressure ulcers, risk level, decision trees, interpretability, classification

## Abstract

Pressure ulcers carry a significant risk in clinical practice. This paper proposes a practical and interpretable approach to estimate the risk levels of pressure ulcers using decision tree models. In order to address the common problem of imbalanced learning in nursing classification datasets, various oversampling configurations are analyzed to improve the data quality prior to modeling. The decision trees built are based on three easily identifiable and clinically relevant pressure ulcer risk indicators: mobility, activity, and skin moisture. Additionally, this research introduces a novel tabular visualization method to enhance the usability of the decision trees in clinical practice. Thus, the primary aim of this approach is to provide nursing professionals with valuable insights for assessing the potential risk levels of pressure ulcers, which could support their decision-making and allow, for example, the application of suitable preventive measures tailored to each patient’s requirements. The interpretability of the models proposed and their performance, evaluated through stratified cross-validation, make them a helpful tool for nursing care in estimating the pressure ulcer risk level.

## 1. Introduction

Pressure ulcers (PUs) [1] are injuries to the skin and underlying tissues, mainly caused by prolonged pressure. These are common on the skin covering the bony prominences of the body, such as the elbows, hips, or tailbone. PUs can cause swelling, warmth, and pain in the affected area, among other symptoms [2]. They tend to heal slowly, and, if left untreated, they can progressively damage deep tissues (including muscles and bones), making them a significant health problem. The impact of PUs extends beyond patients, affecting their families and overloading the resources of the healthcare and socio-health systems [3,4]. Because of this, PUs are considered to be adverse outcomes in clinical practice and need to be avoided.

The prevention of PUs should be, therefore, a priority for nurses, healthcare professionals, and organizations around the world [5]. Nevertheless, the economic cost in healthcare systems of PU prevention measures for patients at risk is usually lower than the cost of other treatments [3,6]. Despite the scientific evidence that has been generated over the years to improve the prevention and treatment strategies, patients with PUs or those at risk of developing them do not always receive the most effective interventions. The decision-making in this field depends on the actions of professionals, their knowledge, attitudes, skills, and other external conditions [7,8]. This variability in nursing practice, influenced by individual choices, highlights the importance of efficient interventions in PU prevention [8].

These factors have led to a large body of literature focusing on the application of techniques for the estimation of PU risk, such as risk assessment scales [9,10] or data science approaches [11,12,13]. For instance, the Norton scale [14] consists of five parameters (physical condition, mental state, activity, mobility, and incontinence), while the Braden scale [9], the most widely used tool in clinical practice, considers six factors (sensory perception, moisture, activity, mobility, nutrition, and friction/shear). Other examples include the COMHON index [15], designed for intensive care, consisting of five items (conscious level, mobility, hemodynamics, oxygenation, and nutrition) and the CALCULATE scale [10], which consists of a total of eight items (too unstable to turn, impaired circulation, dialysis, mechanical ventilation, long surgery, low protein, fecal incontinence, and immobility).

Among the data science approaches, Ting and Garnett [11] conducted a systematic review to evaluate the impact of e-health decision support technologies on the occurrence of PUs. The review highlighted the usability and accuracy of these methods in improving the clinician adherence to prevention practices and reducing healthcare costs. On the other hand, Lee et al. [13] employed data-mining techniques, such as neural networks or support vector machines, to identify the risk factors and discriminate among the different stages of PUs. Raju et al. [16] created models using logistic regression and random forest, among other classification methods, to predict the presence of PUs using different demographic, laboratory, and Braden variables, whereas Moon and Lee [17] considered data with hundreds of variables on patients in long-term care facilities to create decision trees that predict the occurrence of PUs. In addition to these, there are other studies that have also employed different data science methods to create classification models related to the occurrence of PUs [18,19,20].

Despite the abundance of research in the field, and given the inherent complexity, many studies consider numerous variables, often time-consuming or costly, or utilize models that lack interpretability for nursing professionals, thus hindering their ability to understand the rationale behind the model’s decisions. Furthermore, some approaches only distinguish between the presence/absence of PUs without distinguishing between different risk levels, limiting the depth of insight into the patient’s condition. This paper aims to address these gaps in the literature, with a primary focus on enhancing the interpretability of the models for nursing professionals, allowing for the classification of different risk levels of developing PUs using few and easily observable variables. Thus, this research considers tackling several limitations of the Braden scale by offering certain improvements regarding PU risk assessment. By simplifying the patient variables to those most easily and quickly measurable in practice, the variable measurement and differentiation of PU levels will become more efficient. The interpretability granted to the proposed models will represent an added value by providing a deeper understanding of the underlying factors contributing to PU risk, enabling quick consultation by nursing professionals. Additionally, the study will be designed so that the presented models provide the probability of developing each of the levels of PUs and other relevant data, such as the frequency of similar risk profiles in clinical practice. This additional capability will allow for a more comprehensive and detailed assessment of PU risk, which is not achieved with the Braden scale alone. Therefore, this study will not only simplify the application of the Braden scale but also enrich it by providing more complete and valuable information for clinical decision-making.

In this context, a better understanding of the risk level associated with PUs could enable healthcare professionals to make informed decisions and avoid counterproductive practices. Knowing the risk level of PUs is important for nursing praxis as it may enable various applications for prevention and treatment. One of the main preventive measures against the development of PUs is the use of special pressure management surfaces [21,22], such as beds, mattresses, and cushions. The selection of the appropriate support surfaces based on the risk level may be relevant for the prevention of PUs since it allows a distribution of pressure adapted to the needs of each patient [23]. For example, the Spanish national group GNEAUPP [24], which is devoted to the study and advice on PUs and chronic wounds, proposes assigning support surfaces to patients based on four levels of PU risk: no risk, mild risk, moderate risk, and high risk. Consequently, patients without risk of PUs may benefit from static mattresses, while high-risk patients may require additional resources such as replacement mattresses, flotation systems, or fluidized beds [24]. On the other hand, it could also be important to prioritize the allocation of resources based on the risk levels in healthcare facilities facing limitations. The identification of high-risk patients makes it possible to allocate resources, such as support surfaces, in order to optimize their use and guarantee efficient care. Risk levels can also be used to implement other personalized preventive strategies, such as promoting regular repositioning or encouraging the appropriate nutrition and hydration [25]. Additionally, monitoring high-risk patients may allow for the early detection of PUs through periodic examinations, enabling prompt interventions to prevent progression.

The main aim of this paper is to propose interpretable prediction models that assist nursing professionals to estimate the risk level of PUs in each patient. For this purpose, data from 16,215 patients in Granada (Spain) have been collected, focusing mainly on indicators that are quickly identifiable in practice by nursing professionals, such as mobility, activity, and skin humidity [4]. The data present a common problem in nursing classification datasets, such as the presence of class imbalance [26]. In order to address this issue, the dataset is preprocessed and various oversampling configurations are studied to determine the best one to enable the improvement of the data quality before modeling [27]. From the preprocessed data, a classification model based on decision trees has been created using the well-known *Classification and Regression Trees* (CART) [28] algorithm with the aim of predicting the risk level of developing PUs in each patient. The model’s classification performance is evaluated using a stratified cross-validation scheme [29].

In order to enhance the interpretability and practical use of the built model, this research proposes a tabular visualization format for the classifier. It is essential to note that model interpretability, often achieved through *Explainable AI* (XAI) techniques [30], is crucial when dealing with medical applications as it provides deeper insights into the data being processed and the model’s decisions. Consequently, various XAI tools have been employed in the medical domain for diagnostics. For example, *Gradient-weighted Class Activation Mapping* (Grad-CAM), which has been applied in medical areas such as autism spectrum disorder [31] and brain tumor detection [32], uses gradient information from a CNN-based architecture to create a map highlighting the important regions in an image based on the classification. *Local Interpretable Model-agnostic Explanations* (LIME) is another notable XAI method that provides a local estimation for interpreting individual predictions, illustrating the impact of each feature on the model’s outcome, and has been applied to conditions such as Alzheimer’s and Parkinson’s diseases [33,34]. *SHapley Additive exPlanations* (SHAP) allows for interpreting machine learning predictions by visualizing the importance of input variables (e.g., tabular data or pixels) and has been applied in areas such as bloodstream infections [35] and chronic kidney disease [36]. Decision trees, such as CART, have also been employed in other studies to explain more complex models [30]. In this paper, combining CART with tabular visualization techniques leads to a deeper understanding of the model decisions regarding the potential risk of developing PUs. Incorporating these types of approaches into the research can enhance the model’s interpretability and, in turn, increase its potential impact in medical applications.

The remainder of this paper is organized as follows. First, the dataset and the methods used for the construction of the classification models are described. Then, the visual resources for estimating the risk level of PUs are presented. Finally, the results obtained are discussed and the conclusions of this work are summarized, highlighting its main findings.

## 2. Data Collection and Methods

This section describes how the data collection process used in this paper was carried out, as well as the methods used to build the classification models. Thus, the data gathering procedure and the characteristics of the final dataset are presented initially, followed by a description of the algorithms involved in building the classifiers.

### 2.1. Dataset Description

The study associated with this research was conducted in accordance with the principles outlined in the 1975 Declaration of Helsinki [37] and received approval from the *Clinical Research Ethics Committee* of the Andalusian Public Health System in Spain. Data collection utilized the chronic wound registration system (SIRUPP), integrated into the *Diraya* health history application provided by the same health system. Inclusion criteria comprised immobilized patients of both genders, aged 65 years and above, residing in private homes or socio-health centers affiliated with the Clinical Management Units of the Granada-Metropolitan Health District. A patient was considered immobilized if they spent the majority of their time in bed and required assistance to leave it, or if they had significant mobility limitations preventing them from leaving their home except under exceptional circumstances. Excluded were patients who refused to participate, those with cognitive impairment hindering comprehension of the study’s purpose, and terminally ill patients. For patients with cognitive impairment, legal guardians were required to understand the study’s purpose and provide written consent on their behalf. Consequently, the study was conducted within a randomized sample of 16,215 patients meeting the inclusion criteria, drawn from a total population of over 697,000 individuals within the age range considered. The mean age of the participants in the sample was 84.13 ± 9.42 years, with 30.2% being men and 69.8% women.

Three indicators were analyzed for each of the patients under study: the degree of mobility, the degree of activity, and the degree of moisture in the skin. These characteristics were specifically chosen for two main reasons:

1. They have shown significant impacts on the development of PUs in previous works in the literature [38,39]. Thus, exposure to prolonged pressure and shear forces is often associated with decreased activity and mobility, which are known to be primary causes of PUs [40]. Indeed, impaired mobility and activity in patients are increasingly recognized as strong risk factors for the development of PUs [38,39]. Additionally, moisture on the skin can exacerbate the effects of pressure, leading to tissue damage and ulceration. Finally, it should be noted that nurses often depend on their clinical judgment regarding these indicators to start preventive interventions for PUs [41].
2. Even though numerous variables could be considered for predicting the risk of PUs, such as sensitivity, friction, shear, and other metrics derived from medical devices [16,17,18], they can sometimes be costly to obtain. Some of these variables may not be easily observable or measurable in all clinical settings. Furthermore, considering a multitude of variables may affect the interpretability of the models obtained, which would contradict the main aim of this research. The indicators considered in this paper are known for their quick and straightforward identification by nursing professionals [38,39]. This fact will enable the design of a highly interpretable PU risk assessment tool using variables that can be readily recognized without the need for time-consuming procedures or additional resources.

Following the recommendations of risk levels of PUs provided by GNEAUPP [24], the risk level for each patient was determined using the Braden scale [9]: patients with a score of 19 or more are labeled as *no risk*, patients with a score between 15 and 18 as *mild risk*, patients with a score between 13 and 14 as *moderate risk*, and, finally, patients with a score of 12 or less are labeled as *high risk*. A descriptive analysis of the input indicators (*skin moisture*, *activity*, and *mobility*) and the output class (*pressure ulcer risk*) is shown in Table 1.

The results in Table 1 show that most of the patients in the study have a *mild risk* of suffering from PUs (43.50%), whereas the highest risks occur in 29.54% of them. Thus, the sample distribution across various risk levels illustrates a diverse range of patients with some risk of developing PUs, excluding the *no risk* category. This distribution, involving 73.04% of patients at different risk levels, aligns with the main goal of this research of predicting various PU risks, making up a substantial part of the dataset. While these percentages may vary due to their origin from real data, they contribute to a comprehensive representation of each class and the robustness of the classification models developed for PUs prediction. On the other hand, it is important to emphasize the significance of retaining the *no risk* category in the final dataset, constituting 26.96% of the samples. In clinical practice, scenarios where there is an absence of risk are valid and must be considered to ensure the practical utility and real-world applicability of the models developed. By incorporating the *no risk* category, our aim is to provide comprehensive tools that reflect the spectrum of PU risk scenarios encountered by healthcare professionals.

Regarding the indicators, skin moisture seems to be a recurring theme since 60.71% of patients present it, at least occasionally. The activity of the patients is also characterized mainly by walking occasionally (44.05% of observations), although cases in which most of the time they are saddled (26.20%) or bedridden (10.27%) are also frequent. The patient’s mobility is usually very limited (44.35%), which may be another influential factor in the appearance of PUs. Finally, it is important to note that, while each indicator may have its individual functionality in the treatment of PUs, the goal of this research is to create models to discern the risk level of developing PUs based on them. To achieve this, it is necessary to consider the combined use of all these indicators to make these predictions with a certain level of classification performance. The incorporation of multiple indicators aligns with the complexity of real-world clinical scenarios, ensuring that the models account for diverse factors contributing to the prediction of PU risk levels.

### 2.2. Methodology for Building the Decision Tree Models

In order to create the decision tree models that allow estimating the risk level of suffering from PUs based on the indicators of skin moisture, activity, and mobility, a methodology consisting of two main stages is designed:

1. *Data preprocessing*. Before building the final model to predict the risk level of PUs, the dataset is preprocessed to improve the quality of the information used by the classification algorithms in the subsequent phase. This stage primarily focuses on mitigating the issue of class imbalance [42] in the original dataset as the distribution of classes is significantly uneven.
2. *Model building*. After preprocessing the data, we proceed to create the classification model, which enables the estimation of the risk level for each patient in developing PUs. The model is built using a decision tree generator algorithm [28,43], whose output is easily interpretable and applicable in a multitude of contexts.

**Data preprocessing.** As shown in Table 1, the dataset obtained is characterized by being imbalanced [26]. This fact indicates that the data present an unequal distribution among classes [27], implying that the percentage of observations at each PU risk level is quite uneven. For example, the class *high risk* represents 13.07% of the observations, whereas the class *mild risk* represents 43.50%. In classification datasets, this problem may involve that the models that are built from the data are more prone to predict the majority classes while reducing the importance of the minority ones. In order to avoid the negative effect of imbalanced data in the construction of classifiers, it is common to apply preprocessing techniques known as resampling methods [27,42]. These aim to balance the number of observations between the different classes. Thus, once the data are preprocessed, classifier construction algorithms can be applied more reliably.

In order to balance the number of observations in the minority classes, the *random oversampling* [42] technique is used. It is a simple but efficient resampling method that is commonly used with imbalanced classification data with categorical attributes. This mainly consists of choosing a minority class and replicating samples of such class to increase its size. For example, in order to balance the class *high risk*, random observations from this class are iteratively selected and replicated until the same number of observations from the majority class (*mild risk*) is reached.

An important aspect to consider when dealing with imbalanced classification problems is the number of classes in the dataset. In binary problems, it is often sufficient to oversample the minority class to match the size of the majority class, which provides good results in most cases [44]. However, in the case of multi-class problems, previous works have shown that it is interesting to analyze the classes and samples to oversample [27]. Therefore, this research studies different oversampling configurations by preprocessing different combinations of classes, aiming to select the configuration that achieves the greatest improvement in data quality for building the decision trees. Each configuration involves preprocessing some of the minority classes in the dataset, that is, the classes *high risk* (13.07%), *moderate risk* (16.47%), and *no risk* (26.96%), until they reach the size of the majority class (*mild risk*). This procedure will allow minority but relevant classes to be more representative within the dataset for building the classification model later.

**Model building.** After balancing the data, a decision tree building algorithm [43,45] is used to create the classification model. There exist a multitude of classification approaches based on the creation of decision trees, such as *random forest* (RF) [46], XGBoost [47], or bagging methods [48]. Among them, this research uses the CART [28,43] algorithm, which can be considered the basis of many other decision tree techniques, due to two main reasons: *(i)* it is widely used in other medical research works employing data science techniques [49,50,51]; and *(ii)* it is easily interpretable, unlike other ensemble-based models like the aforementioned RF or XGBoost [46,47].

The classifier created by CART is represented as a binary tree. Each node in the tree involves a single input attribute (that is, *skin moisture*, *activity*, or *mobility* in our case), and its branches show the values this variable can take (that is, its different modalities shown in Table 1). Leaf nodes contain the output class that will be used to perform PU risk level prediction. In order to create this binary tree, CART divides the attribute space into different regions, allowing the observations to be categorized into different classes. These regions are defined using a greedy scheme based on a binary recursive partitioning, which searches all the attributes for the value that most reduces the impurity in the nodes (the level of mixing in the class labels of the observations in each of them) [28]. For this, different metrics can be used, such as the deviation function or the Gini index [28], considered in this research.

As a result of the recursive partitioning, CART repeatedly divides the dataset according to impurity reduction until a stopping criterion is satisfied. Among the most usual stopping criteria is considering the minimum number of observations of a node to be partitioned. If the amount of data in a node is not enough, the split can be stopped. Another common procedure is applying a pruning procedure to the tree that allows removing some of the nodes, making the final model more interpretable and reducing its overfitting to the training data. In this context, it is usual to use a complexity parameter that penalizes the tree for having too many splits. This research considers 20 observations per node in the stopping criterion and a complexity parameter 0.01.

The classification performance of the decision tree model is estimated using two metrics: accuracy and geometric mean. Accuracy is a commonly used measure in standard classification tasks, whereas the geometric mean is particularly suitable for imbalanced classification problems. The use of both metrics provides a comprehensive evaluation of the performance of the model across different aspects. In order to ensure robustness, the evaluation is conducted by averaging the test results of 10 runs of a 10-fold cross-validation (10-fcv) [29].

## 3. Interpretable Models for Pressure Ulcer Risk Level Prediction

This section presents the classification models built from the data. First, the different oversampling configurations are studied, as well as their impact on the classification performance, in order to select the best preprocessing approach. Then, the decision tree generated by the CART algorithm is analyzed. Finally, a new way of visualizing the information of the classification model in the form of a table is proposed, aiming to provide a more straightforward and interpretable representation for healthcare professionals.

### 3.1. Analysis of Oversampling Configurations

This section presents the results obtained from different oversampling configurations and their impact on the classification performance. Table 2 provides the oversampling configurations for each risk level and the corresponding values of accuracy (ACC) and geometric mean (GM) for the decision trees using 10 runs of a 10-fcv. The *Oversampling* rows indicate whether each class (*no risk*, *mild risk*, *moderate risk*, and *high risk*) is oversampled or not.

The analysis of the results in Table 2 shows that most of the oversampling configurations achieve relatively high accuracy scores, ranging from 0.7000 to 0.7378. This finding indicates that the decision trees built from oversampled data provide promising results in predicting the risk levels of PUs. However, some oversampling configurations, such as those considering *high risk* with oversampling and *moderate risk* without oversampling, obtain a geometric mean of 0. This fact suggests that these configurations encounter difficulties in effectively addressing the imbalanced nature of the dataset. On the other hand, the preprocessing configurations including the oversampling of the minority classes (both *moderate risk* and *high risk*) achieve the highest geometric mean scores (0.7333 and 0.7356), which indicates that simultaneously oversampling the two highest risk levels of PUs has a positive impact on the classification performance.

Table 2 shows that the optimal preprocessed dataset involves oversampling each minority class—*no risk* (26.96% of the samples), *moderate risk* (16.47%), and *high risk* (13.07%)—to match the size of the majority class—*mild risk* (43.50%). Consequently, this oversampling configuration is chosen for use in the following sections when building the decision trees. Thus, the final dataset maintains an identical sample proportion for each class, with each class representing 25% of the total training set samples for model creation. This procedure implies that all the classes receive equal importance during decision tree construction. It is important to highlight the results of the GM in Table 2, defined as the *c*-th root of the product of the *sensitivity* for each class $\lambda_{1},\ldots ,\lambda_{c}$ in a *c*-class problem. The GM result of the decision tree is 0.6402 for the dataset without preprocessing (the first configuration in Table 2). However, following the equalization of the class proportions during preprocessing (the last configuration in Table 2), the GM value experiences a notable increase to 0.7356. This improvement is substantial compared to not considering any preprocessing, showing the importance of addressing the class distribution problem before the model creation.

### 3.2. Decision Tree for Pressure Ulcer Risk Level Estimation

**Figure 1:**
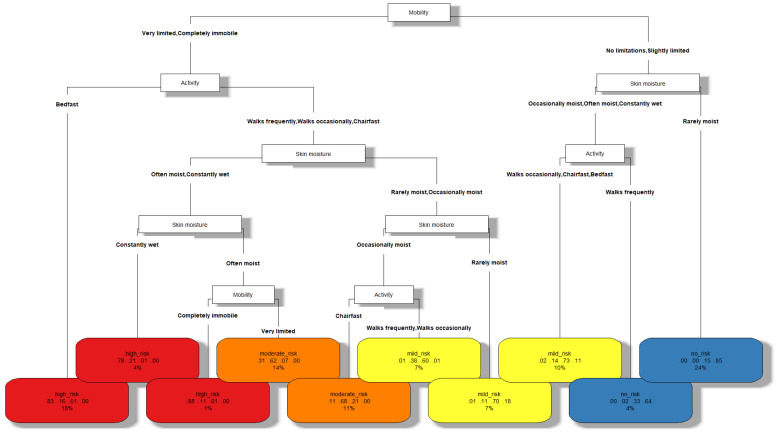
Decision tree for pressure ulcer risk level estimation.

The decision tree built with CART distinguishing among the different risk levels of PUs is shown in Figure 1. As shown in Table 2, its classification accuracy is 0.7378 and its geometric mean is 0.7356. These scores, considering the complexity of the problem addressed, the ease of evaluation in practice of the indicators used, and the interpretability of the resulting model, demonstrate a competitive performance. The elements that are part of this model are described below:

- Each node in the tree represents a characteristic of the patient (*mobility*, *activity*, or *skin moisture*), whereas the leaves of the tree (at the bottom level) represent the different risk levels of PUs (*no risk*, *mild risk*, *moderate risk*, or *high risk*).
- The branches of the tree that depart from each node *A* represent the values that the characteristic *A* of the patient can take.
- In the leaf nodes, a label with the risk level of PUs is found in the first row. A different color has been associated with each of them: blue for *no risk*, yellow for *mild risk*, orange for *moderate risk*, and red for *high risk*.
- The second row of each leaf shows four numbers in the interval [0,1]. These can be viewed as the confidence of the model for classifying the observations regarding that leaf into the different classes in the order of *high risk*, *moderate risk*, *mild risk*, and *no risk*.
- The third row of each leaf represents the percentage of the total observations classified in that leaf, which can be viewed as the frequency of occurrence of the corresponding patient profile in practice.

Note that the consultation of the confidences and the percentage of observations in each leaf node by the healthcare professional can provide important additional information when making the final decision in each case. For example, the sixth leaf node (starting from the left) with a *mild risk* has a frequency of occurrence of 7%, which can be considered as an indicator of dealing with a relatively common patient profile in practice. On the other hand, it can be observed that, although the patient profile corresponding to this leaf probably has a *mild risk* of suffering from PUs (confidence 0.6), it must be taken into account that a *moderate risk* is also highly possible (confidence 0.38).

In order to use the tree, the nursing professional starts from the root node (at the top of the tree) and descends through its branches until reaching a particular leaf. Going through each node, we have a characteristic, and, through each of its branches, the values that it must meet. Finally, the leaf nodes indicate the class corresponding to the patients with that profile. Thus, observing the classification model, the characteristics of the following PU risk profiles can be distinguished:

1. *Characteristics of patients without risk of pressure ulcers.* According to the decision tree, these patients are characterized by mobility with no limitations or are slightly limited. Also, either their skin is rarely moist or they walk frequently.
2. *Characteristics of patients with mild risk of pressure ulcers.* If the conditions of skin moisture and activity of the previous point are not met, the patients have a mild risk of suffering from pressure ulcers. On the other hand, when the mobility is quite limited (very limited or completely immobile) but the patients are not bedridden and their skin is rarely moist, they are also at mild risk. This risk is also present if they are not saddled or bedridden and their skin is occasionally wet.
3. *Characteristics of patients with moderate risk of pressure ulcers.* If patients’ mobility is very limited and their skin is often moist (but they are not bedridden), they are at moderate risk for pressure ulcers. On the other hand, patients with very limited mobility or completely immobile, who are saddled and their skin is occasionally moist, are also at this risk level.
4. *Characteristics of patients with high risk of pressure ulcers.* Patients with very limited mobility or completely immobile, who are bedridden, or whose skin is constantly wet are at high risk of pressure ulcers. Also, if they are not bedridden and their skin is slightly less moist (that is, often moist) but they are completely immobile, they are also at high risk.

The study of the aforementioned profiles in clinical practice makes it possible to identify the main characteristics of the patients at each level of PU risk based on the indicators studied, which are easily recognizable by nursing professionals through the direct observation of the patient. In turn, this information can potentially be used as a complement to the experience of nurses in making decisions related to these patients.

### 3.3. A Tabular Visualization Resource to Increase Interpretability

**Figure 2:**
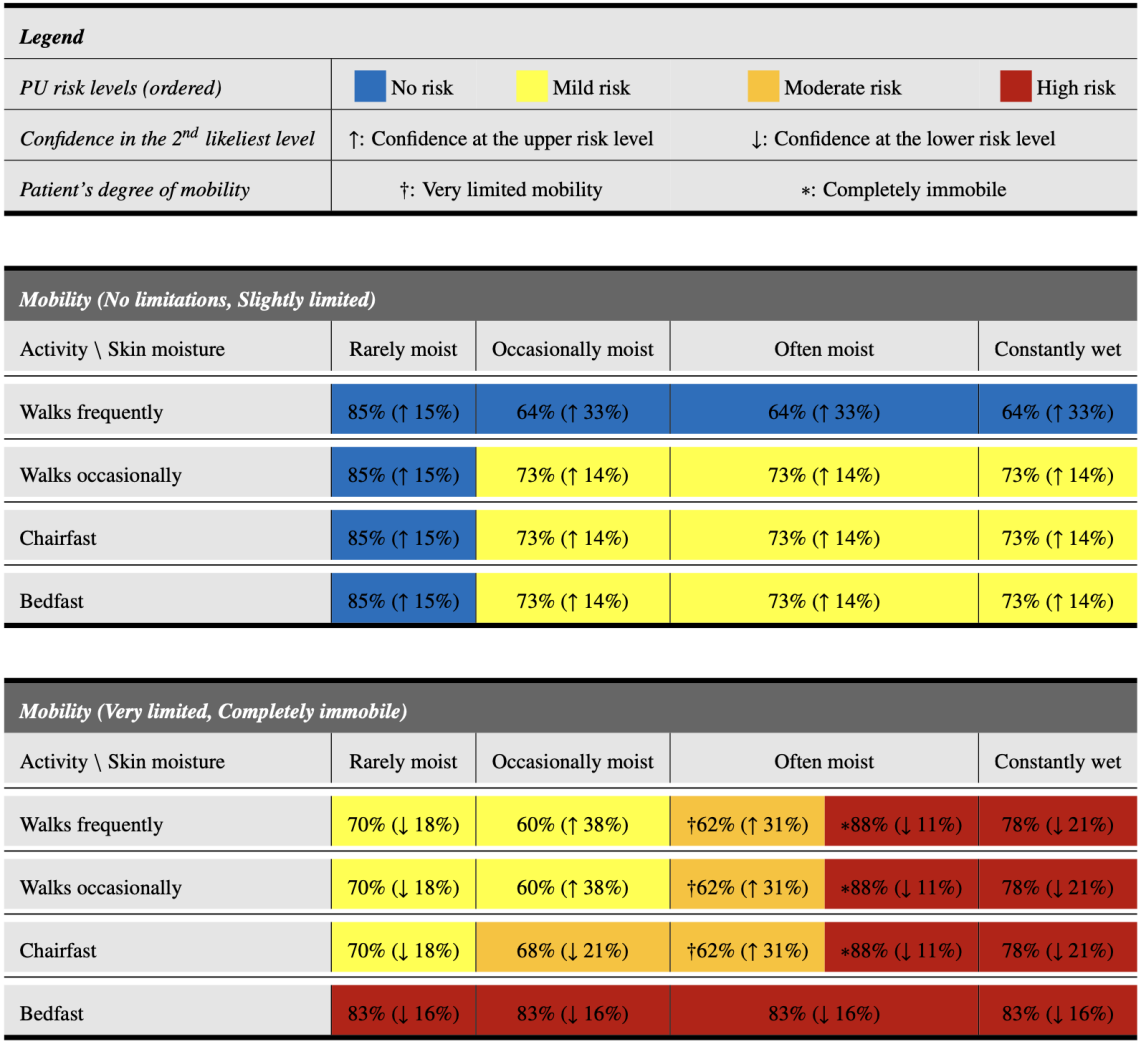
Tabular visualization model to determine the risk level of pressure ulcers. The color of each cell indicates the most probable risk level: blue for *no risk*, yellow for *mild risk*, orange for *moderate risk*, and red for *high risk*. In the lower table, † is used for cells that refer only to patients with very limited mobility, whereas * is used only for completely immobile patients.

The use of instruments, such as the one previously presented, can help nursing professionals make decisions on PU risk level estimation in less time since they are based on quickly identifiable characteristics. This section proposes a novel resource, in tabular form, which aims to further simplify and speed up the decision-making process. This resource, which is based on the information shown in Figure 1 but in a more interpretable way, is presented in Figure 2. It has the following characteristics:

- Excluding the legend at the top, it is divided into two main parts, depending on the mobility of the patient. The upper part focuses on patients with acceptable mobility (no limitations or slightly limited), whereas the lower part focuses on patients with greater mobility problems (very limited or completely immobile).
- Within each part, it is possible to easily identify the level of activity of the patient (in the rows), as well as the level of moisture of the skin (in the columns). Thus, all the characteristics that determine the patient’s profile are determined.
- Once the patient’s profile has been identified, the corresponding cell in the table provides information on their risk level of developing PUs. Its color indicates the most probable risk level: blue for patients with *no risk*, yellow for patients with *mild risk*, orange for patients with *moderate risk*, and red for patients with *high risk*.
- Within each cell, there is also a main percentage (outside the parentheses). This indicates the confidence of the classification model that the patient has the level of PU risk indicated by the color.
- Finally, within each cell, there is also a second percentage in parentheses, accompanied by an arrow. This percentage indicates the confidence in the second most likely risk level, while the arrow (↑ or ↓) indicates if this second level is the upper (↑) or lower (↓) level with respect to the main level expected for that profile. The risk levels are considered in the order of *no risk*, *mild risk*, *moderate risk*, and *high risk*.

As can be appreciated, the tabular model presented in this section enables a rapid categorization of the PU risk levels simply by identifying the appropriate row and column to determine the patient’s profile. In addition, it maintains useful information that can be employed by nurses, such as the confidences in the most likely PU risk levels based on the profile. Thus, for example, among patients with very limited mobility or completely immobile who walk frequently, both those with rarely moist and occasionally moist skin have a *mild risk* of PUs. The former have a 70% probability of being at this risk level, with a tendency to a *no risk* level (18%). However, in the patients with occasionally moist skin, the probability of being at the *mild risk* level is lower (60%), and there is a high tendency to present a *moderate risk* level of PUs (38%).

## 4. Discussion

Decision support models that aid nursing professionals in assessing the risk level of PUs for each patient can facilitate their work. This paper has proposed models based on decision trees that enable distinguishing four levels of PU risk in patients. This differentiation potentially enables the selection of suitable interventions based on the risk level, such as the use of appropriate special support surfaces, in accordance with expert guidelines on PU prevention and treatment (for example, those provided by the technical document of the Spanish national group GNEAUPP [24]). The use of models that help nursing professionals in this context could save time and resources in their choices and serve as a method to prevent possible unwanted situations that may arise in the future. In addition to presenting these models, one of the main objectives of this work is to make them highly interpretable by nurses. To this end, the models have been developed based on characteristics that are easily identifiable by professionals, such as the skin moisture, mobility, and activity of each patient. Furthermore, this work contributes to professional development by providing a comprehensive explanation regarding the use of these decision support instruments.

### 4.1. Implications for Practice

Interpretable classification models, such as the decision trees utilized in this research, hold significant potential for enhancing the clinical practice by providing transparent and understandable decision-making support to healthcare professionals. In this context, where decisions directly impact patient outcomes, the ability to comprehend and trust the reasoning behind the predictive models may be highly relevant. Note that, even though there are algorithms that may offer higher classification accuracy, such as ensemble methods [46,47], they often sacrifice interpretability. This tradeoff implies that the clinical application of such opaque techniques may not be straightforward and efficient across all healthcare settings, in contrast to the models proposed in this research.

Estimating the risk level for PUs in a patient can also influence the clinical practice by enabling healthcare professionals to readily identify individuals at higher risk at an earlier stage, potentially simplifying the decision-making process in this context. The use of these tools underscores the importance of comprehensive training for healthcare professionals involved in data-driven decision-making. While the final decisions ultimately rest with the healthcare provider, the models serve as tools for augmenting the clinical judgment and improving the understanding of complex patient scenarios. As healthcare systems increasingly adopt data-driven approaches, ensuring that clinicians are equipped with the necessary skills and knowledge to effectively utilize these tools becomes imperative.

Finally, interpretable models have the potential to promote greater patient involvement by facilitating clearer communication between patients and healthcare providers, empowering patients in PU prevention and management. Furthermore, implementing predictive models in clinical settings can contribute to achieving equitable care, thereby enhancing the ethical aspects of healthcare practice and ensuring fair treatment for all patients.

### 4.2. Strengths, Limitations, and Assumptions

This research exhibits several strengths that enhance the reliability of the findings. Firstly, the substantial number of participants included in the study contributes to its robust statistical power, thereby enhancing the generalizability of the results. Additionally, a thorough validation process was implemented during data collection to improve the accuracy of the in–out values. The study involved training sessions, maintaining communication with nursing professionals to maintain consistency and reduce the chances of errors or noise.

However, despite the efforts to minimize errors, human error remains a potential source of inaccuracies in the data [52] given the dependence on data entered by nursing professionals. In terms of assumptions, the study considers that the collected data accurately represent the patient population under investigation, without significant bias or missing information. Even though efforts were undertaken to minimize such biases through rigorous data collection and validation processes, inherent limitations in the data collection methods may still exist.

### 4.3. Future Research Directions

In future studies, it is important to continue focusing on enhancing the interpretability of the models in this context, particularly by explaining the reasoning behind the classification system’s decisions. This research was designed to demonstrate a simpler and more efficient and informative approach to obtaining the four main classifications of PU risk levels of the Braden scale. However, the information provided is interpretable and can assist nursing professionals in clinical practice by aiding them in better understanding the challenges they face and making informed decisions. Thus, conducting further studies focused on the interpretability of the models appears to be an area that justifies more attention in future research and could offer significant advantages in this field.

On the other hand, one of the main weaknesses of the commonly used Braden scale is its lack of logical guidance for treatment. This research not only offers a more efficient way to utilize it but could also provide additional insights that contribute to better treatment guidance. Therefore, emphasizing the capabilities of the system introduced in this study is essential for realizing its full potential and guiding future research in this area. For example, while the proposed models allow for the differentiation of four general PU categories (no risk, mild risk, moderate risk, and high risk), similar to the Braden scale, they expand on this by offering different intensities or probabilities for each risk level. This results in up to 10 distinct categories of PU based on patient profiles that consider three easily and quickly observable characteristics. The probabilities of each risk level, along with the frequency of the associated patient profiles in the models, could provide a foundation for designing more targeted treatment strategies in the future based on this information. These expanded models would involve relating each of the 10 categories to tailored treatments and monitoring protocols, for example, considering the anticipated duration of patient immobility, especially in scenarios where minimal interventions are feasible. Therefore, the future investigations could utilize patient data to examine the probability of PU development based on interventions within each category. These treatment strategies, guided by information from the models, will require future clinical studies to validate their efficacy in reducing the occurrence of PUs, which is the ultimate goal of the proposed models.

Conducting further studies could also provide a more nuanced understanding of PU development over time, considering factors such as lesion severity and changes in patient characteristics. Moreover, as inaccuracies can impact the performance, complexity, and construction time of classifiers [53], investigating different data preprocessing techniques becomes relevant. Addressing issues such as class overlapping or noise through approaches like noise filters [54] could contribute to further improvements in the models created. Finally, exploring ethical and legal considerations regarding the implementation of predictive models in healthcare settings, especially concerning the responsible use of patient data and equity, is also noteworthy. Additionally, developing patient engagement strategies to promote transparency and education regarding the predictive models and risk factors for PU prevention would be valuable.

## 5. Conclusions

This research has proposed the use of classification models based on decision trees to predict the risk level of developing PUs. After preprocessing the dataset to overcome the imbalance learning problem, a decision tree model has been built to classify the risk levels of PUs. This model has been created with the well-known CART algorithm, whose resulting tree can be easily interpreted. This allows nurses to know the characteristics of the patient that are indicators of a potential risk level of PUs. The model also provides varying levels of confidence regarding the likelihood that patients within each profile (referring to the characteristics of the variables) may belong to different risk levels, as well as the frequency of occurrence of each of these profiles in clinical practice.

On the other hand, although the presented decision tree is interpretable, its structure and binary character give rise to improvements to increase its practical possibilities. For this reason, this work has also proposed a form of tabular visualization regarding the extracted decision tree with the aim of further improving its interpretability and applicability in real-world situations. This method of presenting the model in tabular form, which maintains information useful to the nurses (such as the confidence in the most probable risk levels), makes it possible to quickly determine the risk level of PUs for each patient simply by locating the row and the column of the table associated with the patient’s profile.

Finally, it is important to mention that the proposed tools aim to address the primary clinical issue, which is not solely risk assessment but rather reducing the risk of PUs. The proposed models provide a logical framework to facilitate the development of treatment measures and monitoring decisions, with the goal of reducing the incidence of PUs. Specifically, the simplicity and interpretability of the models can contribute to faster decision-making, allowing for the assessment of more patients within the same timeframe. This increased efficiency could lead to the earlier detection of PU development and more timely interventions, ultimately reducing the occurrence of PUs. Additionally, the high interpretability of the models and their ease of application will allow nurses to focus on the characteristics on which preventive measures should be centered. Furthermore, other distinctive features of the proposed models, such as the probabilities of the occurrence of each risk level and the frequency of each patient profile, can also help to enable more targeted and specific measures. For example, applying measures to a patient with a *mild risk* of PU, but showing a tendency toward *no risk*, differs significantly from cases where the trend leans towards a higher risk level. This detailed information could enhance the effectiveness of treatment strategies that, even though they will require additional validation studies, will allow interventions to be customized based on the precise risk level, the probability of PU development, and the occurrence of each patient profile in clinical practice.

## Figures and Tables

**Table 1:** Description of the dataset on patients at risk of pressure ulcers, along with the number/ratio of observations at each modality of each indicator.

Indicator	Modality			
**Pressure ulcer risk**	*No risk*	*Mild risk*	*Moderate risk*	*High risk*
*observations*	4371	7054	2670	2120
*ratio*	26.96%	43.50%	16.47%	13.07%
**Skin moisture**	*Rarely moist*	*Occasionally moist*	*Often moist*	*Constantly wet*
*observations*	6371	6147	2762	935
*ratio*	39.29%	37.91%	17.03%	5.77%
**Activity**	*Walks frequently*	*Walks occasionally*	*Chairfast*	*Bedfast*
*observations*	3158	7142	4249	1666
*ratio*	19.48%	44.05%	26.20%	10.27%
**Mobility**	*No limitations*	*Slightly limited*	*Very limited*	*Completely immobile*
*observations*	1357	6458	7191	1209
*ratio*	8.37%	39.83%	44.35%	7.46%

**Table 2:** Evaluation of decision tree classification performance across eight different oversampling configurations. The presence of ✓ indicates intentional oversampling of the respective class until reaching the size of the majority class (*mild risk*), while ✗ signifies that the class remains unaltered without preprocessing.

Oversampling	*No risk*	✗	✗	✗	✓	✗	✓	✓	✓
	*Mild risk*	✗	✗	✗	✗	✗	✗	✗	✗
	*Moderate risk*	✗	✗	✓	✗	✓	✗	✓	✓
	*High risk*	✗	✓	✗	✗	✓	✓	✗	✓
Metric	ACC	0.7215	0.7006	0.7186	0.7255	0.7375	0.7000	0.7186	0.7378
	GM	0.6402	0.0000	0.6801	0.6475	0.7333	0.0000	0.6820	0.7356

## Data Availability

Data available upon request to the authors.
